# Identification of Regulatory Factors for Mesenchymal Stem Cell-Derived Salivary Epithelial Cells in a Co-Culture System

**DOI:** 10.1371/journal.pone.0112158

**Published:** 2014-11-17

**Authors:** Yun-Jong Park, Jin Koh, Adrienne E. Gauna, Sixue Chen, Seunghee Cha

**Affiliations:** 1 Department of Oral and Maxillofacial Diagnostic Sciences, University of Florida College of Dentistry, Gainesville, Florida, United States of America; 2 Interdisciplinary Center for Biotechnology Research, University of Florida, Gainesville, Florida, United States of America; 3 Department of Biology, UF Genetics Institute, University of Florida, Gainesville, Florida, United States of America; 4 Genetics Institute, University of Florida, Gainesville, Florida, United States of America; University of California, Merced, United States of America

## Abstract

Patients with Sjögren’s syndrome or head and neck cancer patients who have undergone radiation therapy suffer from severe dry mouth (xerostomia) due to salivary exocrine cell death. Regeneration of the salivary glands requires a better understanding of regulatory mechanisms by which stem cells differentiate into exocrine cells. In our study, bone marrow-derived mesenchymal stem cells were co-cultured with primary salivary epithelial cells from C57BL/6 mice. Co-cultured bone marrow-derived mesenchymal stem cells clearly resembled salivary epithelial cells, as confirmed by strong expression of salivary gland epithelial cell-specific markers, such as alpha-amylase, muscarinic type 3 receptor, aquaporin-5, and cytokeratin 19. To identify regulatory factors involved in this differentiation, transdifferentiated mesenchymal stem cells were analyzed temporarily by two-dimensional-gel-electrophoresis, which detected 58 protein spots (>1.5 fold change, p<0.05) that were further categorized into 12 temporal expression patterns. Of those proteins only induced in differentiated mesenchymal stem cells, ankryin-repeat-domain-containing-protein 56, high-mobility-group-protein 20B, and transcription factor E2a were selected as putative regulatory factors for mesenchymal stem cell transdifferentiation based on putative roles in salivary gland development. Induction of these molecules was confirmed by RT-PCR and western blotting on separate sets of co-cultured mesenchymal stem cells. In conclusion, our study is the first to identify differentially expressed proteins that are implicated in mesenchymal stem cell differentiation into salivary gland epithelial cells. Further investigation to elucidate regulatory roles of these three transcription factors in mesenchymal stem cell reprogramming will provide a critical foundation for a novel cell-based regenerative therapy for patients with xerostomia.

## Introduction

Salivary acinar cells are responsible for the secretion of water, electrolytes, mucus, glycoproteins, enzymes, and anti-bacterial compounds including salivary peroxidase and lysozyme [1], [2]. Salivary acinar cell death and resulting xerostomia (dry mouth) observed in Sjögren’s syndrome (SjS) and head and neck cancer patients are caused by autoreactive immune cells [3] and radiation therapy. As a consequence, poor quality of life in those patients is inevitable [4]. Current pharmacological therapies to stimulate residual acinar cell function typically fail because glandular damage is already substantial and irreversible by the time patients seek clinical care. Therefore, current treatment options for severe dry mouth patients are mainly palliative and do not improve saliva flow.

Stem cell-based therapies have been applied to repair damaged tissues in various organs. To date, three major types of stem cells have been investigated to regenerate damaged organs; embryonic stem (ES) cells, induced pluripotent stem cells (iPSCs), and adult stem cells [5], [6]. ES cells are pluripotent stem cells derived from blastocysts. iPSC are derived from somatic cells, such as skin or blood cells, that have been reprogrammed back into an embryonic-like pluripotent state by transfecting key transcription factors. iPSCs may become useful in the near future due to their self-renewal capacity similar to embryonic stem cells. However, control of cell differentiation and specific linage development needs to be closely monitored to prevent the formation of teratomas by these cells. Adult stem cells, such as mesenchymal stem cells (MSCs), although not as pluripotent as embryonic stem cells, offer many advantages for the development of restorative treatments. These advantages include but are not limited to their relative accessibility, stable phenotype, tissue compatibility, and immunosuppressive properties.

Bone marrow (BM)-MSCs are multipotent stem cells isolated from bone marrow aspirates [7]. Studies indicate that MSCs can differentiate into osteoblasts [8], chondroblasts [9], adipocytes [10], and even myoblasts [11]. In addition, MSCs can be differentiated into exocrine gland epithelial cells in tissues such as mammary glands, pancreas, liver and salivary glands [12]–[14]. Maria *et al.* have observed that human MSCs differentiate into a salivary gland exocrine cell phenotype through paracrine stimulation during co-culture with parotid or submandibular gland biopsy specimens [15]. Furthermore, allogeneic MSC treatment, injected via tail vein, alleviated symptoms in experimental and clinical SjS [16] and intraglandular transplantation of BM-MSCs ameliorated post irradiation salivary gland damage [17]. However, information on critical regulatory factors responsible for driving MSCs into salivary gland exocrine cells is absolutely lacking to date.

Our current study was to identify differentially expressed regulatory proteins and their temporal expression patterns during mouse BM-MSC transdifferentiation into salivary epithelial cell cells. For our study, mouse MSCs were co-cultured for 1, 3, 5, or 7 days with primary salivary gland cells (pSGCs) isolated from 4–6 week old C57BL/6 (B6) mice and evaluated using 2-dimensional gel electrophoresis (2-DE) proteomics. Expression of potential regulatory factors was also verified by RT-PCR and western blotting. To our best knowledge, our study was the first to discover regulatory factors for MSC transdifferentiation in salivary gland regeneration.

## Materials and Methods

### Animals

C57BL/6J male mice (4–6 weeks of age) were maintained under specific pathogen-free conditions (SPFs) within the Animal Care Services at the University of Florida. The animals were maintained on a 12 h light-dark schedule and provided with water and food *ad libitum*. Both breeding and use of these animals were approved by the University of Florida IACUC. The mice were euthanized according to the American Veterinary Medical Associations’ approved procedures.

### Mouse bone marrow-derived mesenchymal stem cell culture

Mouse MSCs (mMSCs) were purchased from Life Technologies, Inc. The manufacturer isolated mMSCs from bone marrow of C57BL/B6 mice at ≤8 weeks after gestation. Manufacturer reported a purity of >95% cells positive for expression of cell surface markers indicative of mMSCs (i.e. CD29^+^, CD44^+^, CD34^+^, Sca1^+^), and tested their ability to differentiate into osteocytes, adipocytes, and chondrocytes *in vitro*. The cells were cryopreserved at passage eight following *in vitro* expansion. mMSCs were thawed and cultured in our laboratory in T-75 tissue culture flasks containing 15 ml of DMEM/F12 with 10% MSC-qualified FBS and 5 µg/ml gentamycin. The culture flasks were incubated in 5% CO_2_ at 37°C. mMSCs were passaged using 0.05% trypsin-EDTA (Life Technologies, Inc) every 3–4 days when cells reached 80–90% confluence. All experiments used mMSCs with passages between 3 to 7 after thawing.

### Mouse primary salivary gland cell isolation and culture

Mouse salivary gland cells (pSGCs) were carefully prepared to avoid contamination of other types of cells following a published protocol [18]. In short, submandibular glands were washed with 1% (w/v) bovine serum albumin in Hanks’ balanced salt solution. The tissue was cut finely using sterile curved dissection scissors and a surgical blade in a small petri dish. Then, the pieces were subject to two rounds of enzymatic digestion with collagenase II (0.25 mg/ml) (Life Technologies, Inc) and CaCl_2_ (6.25 mM) at 37°C for 40 minutes in a water-bath with gentle mechanical shaking. The cell suspension was filtered with a 100 µm steel mesh and plated on non-coated 60 mm plates at a density of 1.2×10^6^ cells per well. After plating the cells on a petri dish, the dish was manually rotated to concentrate epithelial cells in the middle. The cells in the center were further seeded for culture. Approximately, 3.0×10^6^ to 3.5×10^6^ pSGCs were isolated per one pair of submandibular glands. Isolated pSGCs were cultured in the serum-free Hepato-STIM medium (BD BioCoat) consisted of 500 U/ml penicillin/streptomycin for 12 hrs prior to co-culture with mMSCs in order to equilibrate and stabilize cells into media condition.

### Co-culture of mMSCs and pSGCs

All co-culture experiments were conducted in 6- or 24-well plates containing a 0.4 µm pore size polycarbonate membrane-based transwell insert (EMD Millipore). mMSCs (1.0×10^4^ cells/cm^2^) were seeded on the collagen coated lower chamber of the cell culture plate and incubated in Hepato-STIM media without serum for 12 hr. Once mMSCs were well attached to the bottom of the plate, isolated pSGCs (6×10^4^ cells/cm^2^) were seeded onto the membrane of the transwell insert. Cells in the co-culture system were maintained at 37°C and 5% CO_2_ in a humidified atmosphere without replacing the media and harvested from different time points: 1, 3, 5, and 7 days. Control mMSCs were cultured as described above without co-culturing with pSGCs for each time point.

### Two-dimensional gel electrophoresis (2-DE) and protein staining

Unless otherwise stated, chemicals used in this study were purchased from Sigma Chemical. Cultured MSC cell suspensions in lysis buffer were sonicated for 30 seconds on ice for 5 times and the soluble fractions were collected by centrifugation at 15,000 rpm for 15 min at 4°C. Prior to isoelectric focusing, IPG ReadyStrip 18 cm pH 3–10 (Bio-Rad Laboratories, Inc.) were rehydrated at room temperature for 16 hr in a lysis buffer containing 200 µg of cellular lysates. Isoelectric focusing was performed at 18°C with a current limit of 50 µA/strip. Voltage was increased progressively to a total of 60 kVhr: 1 hr at 100 V, 1 hr at 500 V, 2 hr at 1,000 V, 2 hr at 4,000 V and 10,000 V until the final voltage was reached. The focusing apparatus was an Ettan IPGphor (GE Healthcare Life Sciences). IPG strips were equilibrated for 15 min by gentle shaking in 6M urea, 2% SDS, 5 mM tributyl phosphine, 1M Tris-HCL, and 30% glycerol. Vertical SDS gradient slab gels (12.5%, 18 cm) were used in the second dimension of electrophoresis. The second-dimension gels were overlaid with a solution containing 0.5% agarose, 1 M Tris-HCl, 0.1% SDS, and a trace of bromophenol blue. Electrophoresis was conducted in SDS PAGE gel running buffer at 18 mA per gel at 18°C. The gels were fixed and stained using mass spectrometry-compatible silver stain kit (Thermo Fisher Scientific Inc.).

### In-gel digestion and mass spectrometry

The silver stained gels were scanned using a densitometer at a resolution of 300 dots per inch in the transparency mode. The gel images were analyzed using the Proteomeweaver 2-D Analysis software (Bio-Rad Laboratories Inc.). Statistical comparisons were made using Student’s t-test with statistical significance defined at *p*<0.05. Comparisons were made among groups (10 gels from 5 independent experiments in duplicate per group). Analyses of protein expression profiles in co-cultured samples harvested at 1, 3, 5, and 7 days were performed. After normalization of protein spots, intensities for each time point were transformed by the log_2_ value of the ratio between the experimental and control results at a given time point. Protein spots of interest were manually excised from silver-stained 2-DE gels for in-gel digestion. Gel slices were washed and reduced by dithiothreitor and alkylated by iodoacetamide before tryptic digestion over night at 37°C. Peptide fragments of each spots then was analyzed with LC-MS/MS at UF ICBR core facility. The trypsin digested samples were injected onto a capillary trap (Dionex) and desalted for 5 min with a flow rate of 3 µl/min of 0.1% v/v formic acid. The samples were loaded onto an LC Packing C18 Pep Map nanoflow HPLC column (Dionex). The elution gradient of the HPLC column started at 3% solvent A, 97% solvent B and finished at 60% solvent A, 40% solvent B for 30 minutes for protein identification.

### Protein identification and data analyses

Tandem mass spectra were extracted and data were analyzed using Mascot 2.4 (Matrix Science). Mascot was set up to search against the UniProt *Mus musculus* FASTA database (87,195 entries) assuming the digestion enzyme trypsin. Mascot was searched with a fragment ion mass tolerance of 0.80 Da and a parent ion tolerance of 10.0 PPM. Carbamidomethyl of cysteine was specified in Mascot as a fixed modification. Glu->pyro-Glu of the n-terminus, gln->pyro-Glu of the n-terminus, oxidation of methionine, phospho of serine and threonine and glyGly of lysine were specified in Mascot as variable modifications. For protein identification, we validate MS/MS based peptide and protein identifications from Mascot results using Scaffold v4.0.4 (Proteome Software Inc.). Peptide identifications were accepted if they could be established at greater than 0.8% probability by the Peptide Prophet algorithm [19] with Scaffold delta-mass correction. Protein identifications were also accepted if they could be established at greater than 95.0% probability and contained at least 2 identified peptides. Protein probabilities were assigned by the Protein Prophet algorithm [20]. Proteins that contained similar peptides and could not be differentiated based on MS/MS analysis alone were grouped to satisfy the principles of parsimony from Mascot and Scaffold analyses. Proteins sharing significant peptide evidence were grouped into matched amino-acid clusters against the database.

### Western blot analysis

Following a conventional protocol, aliquots of 20 µg of each sample were mixed with loading buffer (60 mM Tris-HCl, 25% glycerol, 2% SDS, 14.4 mM 2-ME, 0.1% bromophenol blue), separated on 12.5% SDS-PAGE gels, and transferred to a PVDF membrane. Membranes were blocked for 1 hr with 5% non-fat dry milk and incubated with antibodies against α-amylase (α-AMY, salivary specific), muscarinic acetylcholine receptor type 3 (M3R), aquaporin-5 (AQP-5), cytokeratin19 (CK19), ankyrin repeat domain-containing protein 56 (Ankrd56), high mobility group protein 20B (Hmg20b), or transcription factor E2a (Tcf3) (Santa Cruz Biotechnology, Inc.). Membranes were stripped to detect GAPDH expression and incubated with anti-GAPDH antibody from Abcam, Inc. Blots were washed and then incubated for 1 hr at room temperature with horseradish peroxidase-conjugated anti-rabbit or anti-goat antibodies. Bands of antibody binding were visualized by enhanced chemiluminescence western blotting detection system (GE Healthcare Life Sciences), and protein expression levels were normalized to the GAPDH expression level following densitometer analyses (ImageJ; http://rsb.info.nih.gov/ij).

### Total RNA isolation and RT-PCR

Following aspiration of medium after co-culture, cells were lysed directly in the culture dish by adding TRIzol reagent (Life Technologies, Inc.). The samples were prepared following the manufacturer’s instruction. cDNA was synthesized from 2 ug of total RNA using PCR master mix (Promega, Inc.). cDNA samples were subjected to semi-quantitative RT-PCR with gene-specific DNA primers. All PCR reactions were performed under the condition of 25 cycles at 60C° and the sequences of the primers used are provided in Table S1.

### Immunocytochemistry

Co-cultured or control mMSCs grown on collagen-coated coverslips (BD BioCoat) were fixed with 4% paraformaldehyde for 20 minutes at room temperature. pSGCs purified from the submandibular glands were prepared by cytospinning at 250 g for 5 min and fixed as described above for positive control staining. After fixation, the cells were washed, permeabilized, and blocked in PBS containing 0.2% Triton X-100 and 5% fetal bovine serum(FBS) for 30 min at room temperature. Cells were then incubated with goat polyclonal anti-α-AMY (1∶300), rabbit polyclonal anti-M3R (1∶200), rabbit polyclonal anti-AQP-5 (1∶150) and goat polyclonal anti-CK19 (1∶200) in PBS containing 0.1% Triton X-100 and 1% FBS at 4°C overnight. After washing with PBS, the cells were incubated at room temperature for 1 hr with appropriate fluorescence-conjugated secondary antibodies (1∶200 dilution, Molecular Probes) in PBS containing 0.1% Triton X-100 and 1% FBS. Coverslips were mounted and nuclei stained with Vectashield mounting solution containing DAPI (Vector Laboratories, Inc.). Fluorescence was observed under a 20X or a 40X magnification using a Zeiss Axiovert 200M microscope equipped with a Zeiss AxioCam MRm camera and images were obtained from AxioVs40 software (Ver. 4.7.1.0, Zeiss). Positively stained cells that overlapped with DAPI staining in 4 random fields per slide image were counted with a cell counter in a blinded manner following image uptatke at a 10X objective. The results were expressed as the percentage of positively stained cells/total number of DAPI positive cells.

### Statistical analysis

All data with normal distribution are presented as mean ± standard error of over 3 independent experiments. Positive cell numbers and expression levels of proteins or mRNAs were statistically analyzed with one-way ANOVA with Bonferroni post-hoc test using JMP 9.0.1 (SAS institute). p values less than 0.01 or 0.05 were considered as statistically significant. The graphs with statistical analysis were generated by Prism software (GraphPad Software, Inc.).

## Results

### The gross morphology of co-cultured mMSCs resembled that of pSGCs

**Figure 1:**
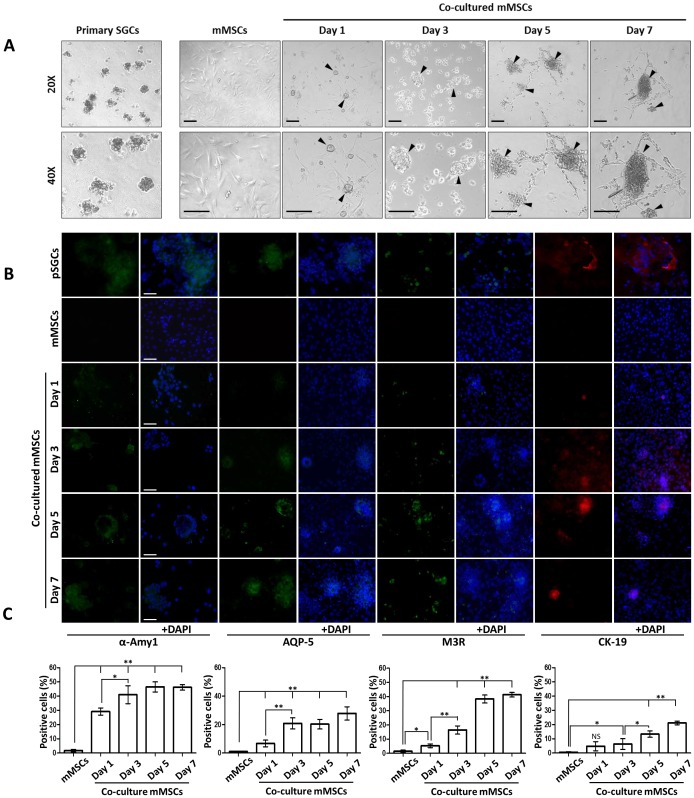
Co-cultured mMSCs resemble primary salivary gland cell morphology and express salivary gland epithelial cell markers. A) Microscope images (at 20X and 40X magnifications) of pSGCs from C57BL/6 mice (first panel), control mMSCs (second panel), and co-cultured mMSCs with pSGCs were shown. Mouse pSGCs showed islet-like cell morphology whereas control mMSCs exhibit typical fibroblast-like appearance. Aggregated cell masses, which resemble islet-like pSGCs, at each time point were indicated by black arrowheads. Co-culture was carried out for 7 days without replacing media. B) Co-cultured mMSCs were positively stained for acinar cell markers, such as a-amylase, and M3R (green color in each column) in a time dependent manner and a ductal cell marker CK19 (red color). Control mMSCs (second row) were negative while cytospinned pSGCs (first row) from the submandibular glands were positive for these markers. The nuclei were stained with DAPI and the column of +DAPI indicates merged images. Scale Bar = 50 µm. C) Co-cultured mMSCs were counted from four independent biological replicates after staining using a fluorescent microscope. Y-axis represents a percentage of positively stained mMSCs for each marker protein at a given time point. Pictures were taken at a 20X magnification. Quantification of cell numbers over time was performed by one-way ANOVA with Bonferroni post-hoc test (*p<0.05, **p<0.01, NS: no significant).

To select the right type of cell culture media that were compatible with both mMSCs and pSGCs, two different types of media without serum were compared. DMEM/F12-glutamax has been used for mMSCs and hepato-STIM media were specific for human pSGC [15]. Both mMSCs and pSGCs maintained their populations in hepato-STIM media without significant cell death as measured by MTT assays for up to 10 days of culture (Fig. S1A). As for co-culture, mMSCs were plated on the collagen-coated glass slide in the culture wells for 12 hours before co-culture and the upper transwell membrane was seeded with pSGCs (Fig. S1B). In this system, only soluble factors can diffuse through the transwell membrane between mMSC and pSGC compartments. During co-culture for up to 7 days, pictures were taken every other day to evaluate mMSC gross morphology (Fig. 1A). mMSCs without pSGCs (control) showed a typical stem cell fibroblast-like morphology. However, co-cultured mMSCs underwent morphological changes to become round as the culture progressed, eventually resembling pSGCs that exhibited an islet-like morphology (Fig. 1A). Round MSCs form clusters at day 3 and formed islets at day 5 with multiple connections/projections reaching out to neighboring islets (Fig. 1A).

### mMSCs expressed salivary gland cell markers as a result of co-culture with pSGCs

Based on the resulting morphological changes in mMSCs following co-culture with pSGCs, we hypothesized that co-cultured mMSCs differentiated into salivary epithelial cells. To test our hypothesis, we performed immunocytochemistry and western blot analysis on co-cultured mMSCs for known salivary gland acinar cell markers, such as α-amylase (α- AMY), muscarinic acetylcholine receptor (M3R), and aquaporin-5(AQP-5) and the ductal cell marker cytokeratin 19 (CK19). Co-cultured mMSCs showed a time-dependent increase in acinar and ductal cell marker protein expression (Fig. 1B and 1C). Co-cultured mMSCs showed dispersed cytoplasmic expression of acinar cell markers of α-AMY and AQP-5 while M3R expression was mainly concentrated in the clustered mMSCs. In addition, the CK19 was primarily expressed in the mMSC aggregates (Fig. 1B). The control mMSCs were negative for these markers while pSGCs showed positive staining as expected.

To objectively evaluate protein expression in co-cultured MSCs, the number of mMSCs expressing α-AMY, AQP-5, M3R and CK19 were counted as the percentage of positive cells following co-culture (Fig. 1C). Percentages of α-AMY-positive mMSCs at all time points were significantly increased when compared to the mMSCs (p<0.01) and then were sustained at around 30% to 45%. Percentages of AQP-5-positive mMSCs were not significantly increased until day 3 and the number of positive MSCs stabilized around 22% for the remaining time points. In addition, percentages of M3R-positive mMSCs increased at day 1 (p<0.05) and maintained over time around 35% at days 5 and 7 (p<0.01). In contrast, percentages of CK19-positive mMSCs did not change significantly at day 1 but expression was increased after 3 days of culture (p<0.05). At day 7, the number of CK19-positive mMSCs was around 20% of the population (Fig. 1C).

**Figure 2:**
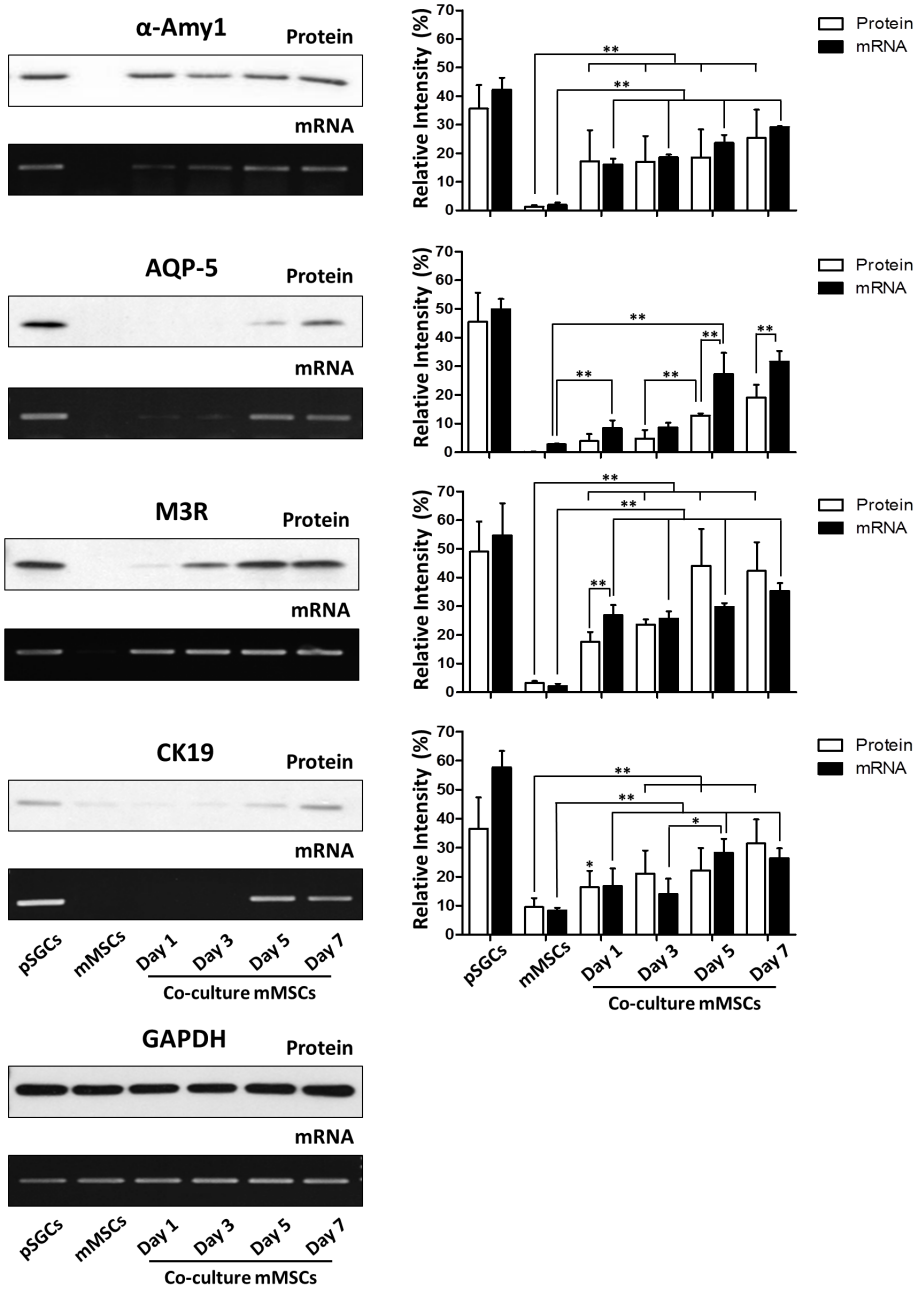
Specific salivary epithelial cell markers were expressed in co-cultured mMSCs, as detected by western blotting and RT-PCR analysis of cell-specific markers. Total protein lysate and mRNA samples isolated from pSGCs from 4 week-old B6 mice were used as a positive control. mMSCs without co-culture was used as a negative control. Acinar markers of salivary specific α-AMY, M3R and AQP-5 were detected in pSGCs and co-cultured mMSCs. Densitometer analyses of the expressed proteins and genes in three independent replicates(*p<0.05, **p<0.01, one-way ANOVA with Bonferroni post-hoc test).

The expression of the salivary epithelial cell markers in differentiated mMSCs was verified by western blot analysis and RT-PCR (Fig. 2) with the lysate of pSGC from 4 week-old B6 mice (n = 8) as a positive control. Similar to the immunocytochemistry results in Fig. 1B, α-AMY and M3R protein expression were significantly increased in the co-cultured mMSCs when compared to mMSCs without culturing with pSGCs (p<0.01, one-way ANOVA with Bonferroni post-hoc test). AQP-5 and CK19 gradually increased their expression over time in differentiated mMSCs, which is also reflected in mRNA expression profiles (Fig. 2) (p<0.05, one-way ANOVA with Bonferroni post-hoc test).

### Proteomics analyses identified differentially expressed proteins in mMSCs during MSC-to-SGC transdifferentiation *in vitro*

**Figure 3:**
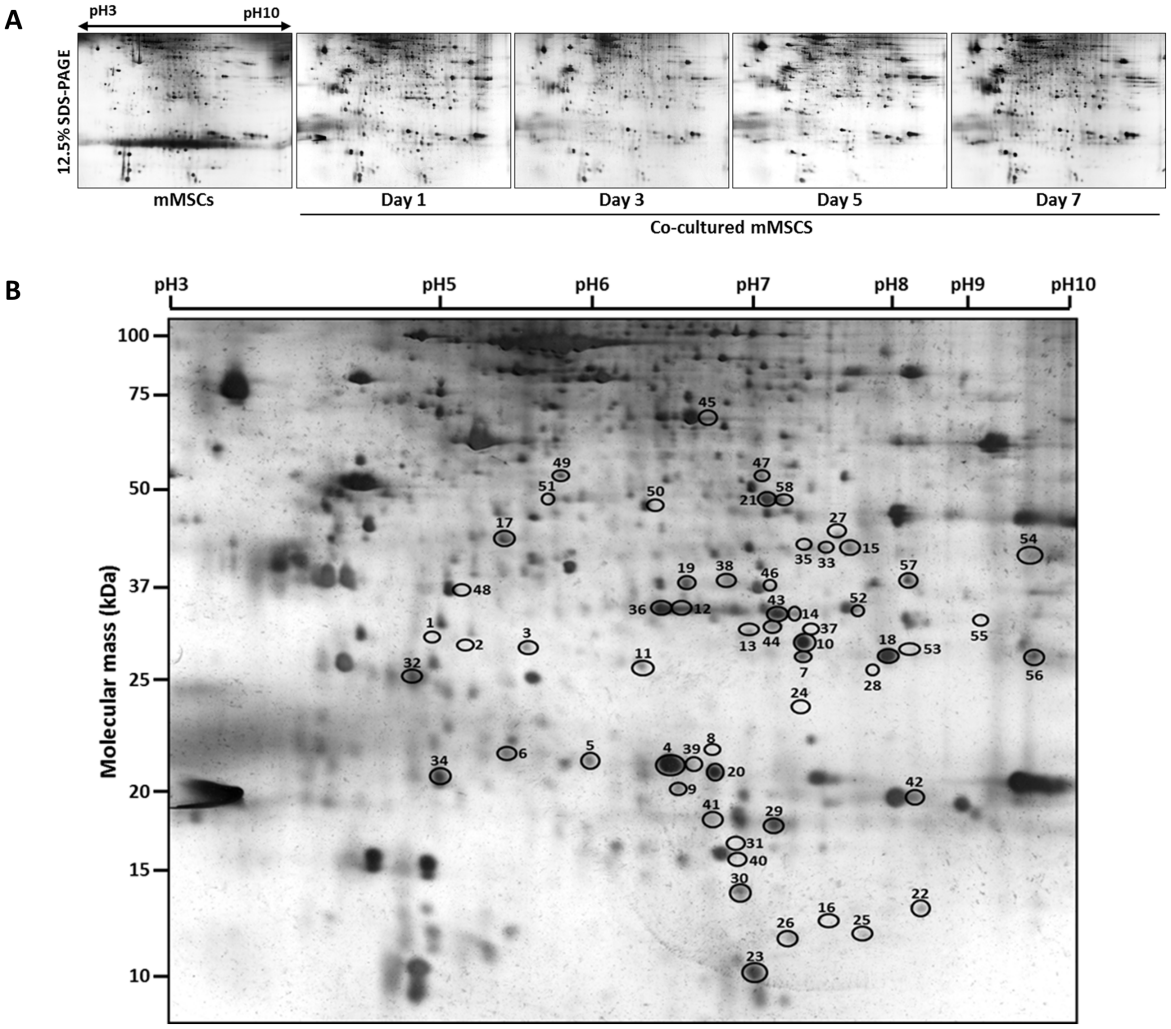
Two-dimensional gel electrophoresis images and spot analysis revealed 58 differentially expressed proteins. A) Following the co-culture of mMSCs with pSGCs for 1, 3, 5 and 7 days, total cell lysates (200 µg) were separated on pH 3–10 linear IPG strips in the first dimension and 12.5% SDS-PAGE in the second dimension. The gels were stained with mass spectrophotometry-compatible silver staining kit. B) According to the data analyses, expression levels of 58 spots (circled) were significantly altered at least by 1.5 fold (p<0.05, one-way ANOVA with Bonferroni post-hoc test). Each of these spots has a specific spot number for database storage and further analysis. Data from five independent experiments (Five gels in duplicate for each time point) were analyzed and the gel figure presented here is from day 7.

To identify regulatory molecules for mMSC differentiation into salivary epithelial cells during co-culture and characterize expression profiles, we applied a proteomics approach of two-dimensional gel electrophoresis (2-DE). The samples prepared from the cultured mMSC cell lysate were separated on a 12.5% polyacrylamide SDS-PAGE gel in pH range 3∼10 for IEF (isoelectric focusing) and visualized by silver staining (Fig. 3A). Spot analysis was performed using Proteomweaver 2-DE Analysis software on five independent experimental data with two gels prepared for each time point. If a spot was not detected in all gels for the same time point, this was considered as a false positive and subtracted from further data analyses. In order to normalize spot intensity in all 10 gels, an absolute intensity value of each spot was divided by the sum of absolute intensity values of all spots. This relative intensity of each spot was then compared with that of each spot in the control (Fig. 3A). More than 271 matched spots ranging from approximately 10 to 100 kDa were detected in control and co-cultured MSC gels. Among these, 58 spots (numbered 1–58) were selected based on the fold change of at least 1.5 (p<0.05) at 1, 3, 5, and 7 days of co-culture (Fig. 3B). To identify these differentially expressed proteins, protein spots were excised, digested, and analyzed by LC-MS/MS and a protein identity was assigned for each spot, based on our reference database of UniProt *Mus musculus*. Molecular weights of these 58 differentially expressed proteins (Table 1) ranged from 10 kDa to over 100 kDa and percent sequence coverage ranged from over 5% to 63%. Two spots (spot#1 and #2) among 58 identified spots did not yield any matched identification from the databank potentially because the amount of protein isolated from the gels may have been insufficient to be analyzed by LC-MS/MS.

**Table 1:** Identification of proteins selected by 2DE analysis.

**Spot # ^A)^**	**UniProt** **Acc. # ^B)^**	**Gene Identification**	**Gene abbreviation**	**# AA ^C)^**	**M.W (kDa) ^D)^**	**calc. PI ^E)^**	**# Pep ^F)^**	**Cov %^ G)^**
**3**	Q9R0Y5	Isoform 2 of Adenylate kinase isoenzyme 1	AK1	194	21.54	5.67	2	7.0%
**4**	P18760	Cluster of Cofilin-1	CFL1	166	18.50	8.09	5	24.0%
**5**	Q4FK36	Destrin	DSTN	165	18.50	8.14	4	30.0%
**6**	A2RSH1	cAMP-specific 3′,5′-cyclic phosphodiesterase 4D	PDE4D	754	86.30	5.07	3	8.1%
**7**	Q9R1P3	Proteasome subunit beta type-2	PSMB2	201	22.90	6.52	2	14.0%
**8**	Q9Z1R9	MCG124046	PRSS1	246	26.10	4.94	3	13.0%
**9**	Q9D967	Magnesium-dependent phosphatase 1	MDP1	164	18.58	6.29	1	8.0%
**10**	Q14AA6	MCG49183	Q14AA6	216	24.36	7.75	2	19.0%
**11**	Q69ZM6	Isoform 2 of Serine/threonine-protein kinase 36	STK36	1316	144.18	5.59	2	5.0%
**12**	P97429	Annexin	ANXA4	319	36.00	5.57	5	15.0%
**13**	O08915	AH receptor-interacting protein	AIP	330	37.50	6.40	5	15.2%
**14**	Q9WTP6	Isoform 2 of Adenylate kinase 2, mitochondrial	AK2	239	26.47	6.96	9	20.0%
**15**	Q60930	Voltage-dependent anion-selective channel protein 2	VDAC2	295	31.73	7.44	5	18.0%
**16**	Q9CPP6	NADH dehydrogenase 1 alpha subcomplex subunit 5	NDUFA5	116	13.36	7.82	3	63.0%
**17**	Q9R059	Four and a half LIM domains protein 3	FHL3	289	31.79	5.80	13	37.0%
**18**	P19157	Glutathione S-transferase P 1	GSTP1	210	23.60	7.69	4	23.0%
**19**	Q9JKX6	ADP-sugar pyrophosphatas	NUDT5	218	23.98	5.34	5	18.0%
**20**	Q6ZWQ5	Sorting nexin 12, isoform CRA_b	SNX12	162	18.90	8.44	3	21.0%
**21**	Q6P5G3	MBT domain-containing protein 1	MBTD1	631	70.67	7.96	2	7.0%
**22**	P26883	Peptidyl-prolyl cis-trans isomerase FKBP1A	FKBP1A	108	11.90	7.89	2	24.0%
**23**	P08207	S100 calcium binding protein A10 (Calpactin), isoform	S100a10	97	11.20	6.77	9	48.0%
**24**	Q08024	Isoform 2 of Core-binding factor subunit beta	CBFB	187	22.03	5.59	2	10.0%
**25**	Q64433	10 kDa heat shock protein, mitochondrial	HSPE1	102	11.00	8.35	2	28.0%
**26**	Q6NTA4	Ras-related GTP-binding protein B	RRAGB	374	43.19	5.99	2	8.0%
**27**	Q564E2	L-lactate dehydrogenase	LDHA	332	36.50	7.61	2	10.0%
**28**	P09671	Superoxide dismutase	SOD2	222	24.60	8.62	4	17.0%
**29**	Q9DAG9	PHD finger protein 7	PHF7	307	35.38	8.95	2	12.0%
**30**	Q8BZW2	Ankyrin repeat domain-containing protein 56	ANKRD56	760	83.54	8.52	3	7.0%
**31**	P62889	Rpl30 protein (ribosomal protein L30)	RPL30	115	12.78	9.65	2	14.0%
**32**	P70296	Phosphatidylethanolamine-binding protein 1	PEBP1	187	23.00	5.40	3	19.0%
**33**	Q8VBV3	Exosome complex component RRP4	EXOSC2	293	32.63	7.06	5	18.0%
**34**	P63242	Eukaryotic translation initiation factor 5A-1	EIF5A	154	16.80	5.24	5	21.0%
**35**	Q99LC5	Electron transfer flavoprotein subunit alpha	ETFA	333	35.00	8.62	4	23.0%
**36**	P68040	Guanine nucleotide-binding protein subunit beta-2-like 1	GNB2L1	317	35.10	7.69	2	12.0%
**37**	Q6ZVL3	PDZ domain actin binding protein Shroom mRNA	Q6ZVL3	889	87.86	9.56	4	18.0%
**38**	Q9DBJ1	Phosphoglycerate mutase 1	PGAM1	254	28.83	6.67	5	26.0%
**39**	P50580	Proliferation-associated protein 2G4	PA2G4	394	43.70	6.41	3	19.0%
**40**	Q3UU43	MCG16489, isoform CRA-a	CHPF2	768	85.65	7.64	2	7.0%
**41**	P10853	Histone H2B (Fragment)	HIST1H2BJ	126	13.94	10.31	4	29.0%
**42**	P17742	Peptidyl-prolyl cis-trans isomerase A	PPIA	164	18.00	7.90	2	16.0%
**43**	Q9Z104	High mobility group protein 20B	HMG20B	317	35.87	9.33	3	12.0%
**44**	O35737	Heterogeneous nuclear ribonucleoprotein H	HNRNPH1	449	49.20	6.30	2	8.0%
**45**	O88712	Isoform 2 of C-terminal-binding protein 1	CTBP1	367	39.80	6.38	16	18.0%
**46**	P67778	Prohibitin	PHB	272	29.80	5.76	2	18.0%
**47**	P60335	Poly(rC)-binding protein 1	PCBP1	356	37.50	7.09	5	17.0%
**48**	O55239	Nicotinamide N-methyltransferase	NNMT	264	29.60	5.27	3	12.0%
**49**	Q9Z0S1	3′(2′),5′-bisphosphate nucleotidase 1	BPNT1	308	33.20	5.54	3	13.0%
**50**	Q91VJ5	Pqbp1 protein	PQBP1	263	30.60	5.86	3	25.0%
**51**	Q9QX98	ptf1a	PTF1A	301	35.60	4.99	3	9.0%
**52**	Q3TZ89	Isoform 2 of Protein transport protein Sec31B	SEC31B	1158	125.63	8.00	2	4.0%
**53**	Q01063	cAMP-specific 3′,5′-cyclic phosphodiesterase 4D	PDE4D	747	84.56	4.79	4	18.0%
**54**	Q61425	Hydroxyacyl-coenzyme A dehydrogenase, mitochondrial	HADH	314	34.40	8.65	9	21.0%
**55**	P08074	Carbonyl reductase [NADPH] 2 (Fragment)	CBR2	244	26.00	9.10	2	5.0%
**56**	E9PWE2	Transcription factor E2a	TCF3	653	67.89	5.94	8	49.0%
**57**	Q6ZPW1	Protein Znf512b	ZNF512B	883	96.58	9.80	7	29.0%
**58**	Q9CPN9	Protein 2210010C04Rik	2210010C04Rik	247	27.09	8.22	2	13.0%

A) Spot number, B) UniProt accession number, C) Number of matched amino-acids, D) Molecular weight, E) Calculated PI (isoelectronic point), F) Number of matched peptides, and G) Coverage percentage.

### Differentially expressed proteins were categorized based on their temporal expression profiles and biological processes

**Figure 4:**
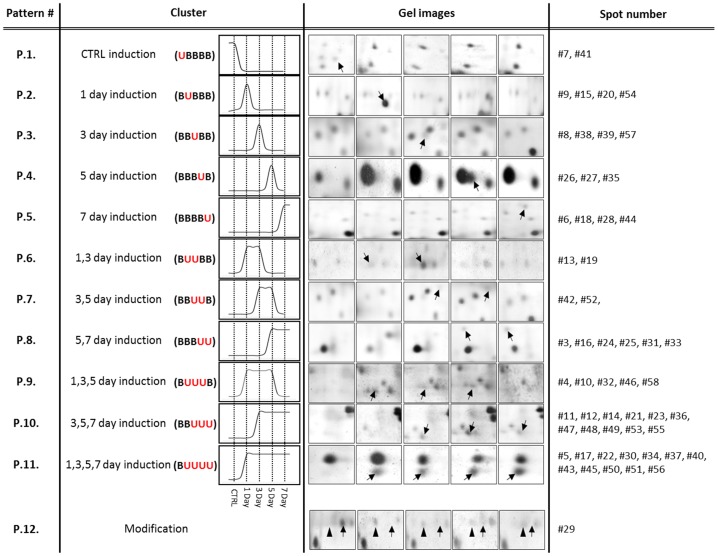
Categorization of 58 spots based on temporal expression profiles. Fifty-eight spots were grouped based on their temporal expression patterns following spot analysis for each time point (B: basal expression; U: up-regulation; M: modification). All identified spots in 2-DE gel are categorized into 12 patterns based on their expression profiles (p<0.05, one-way ANOVA). The examples of spots corresponding to the expression pattern or profile were shown. Black arrows indicate up-regulated spots at each time point. In the pattern #12, black arrows indicate spot was shifted into a different pH location on gels as the culture progresses. Arrowheads indicate increased expression of the same protein during co-culture.

To determine protein expression profiles over time and predict their relative impact on mMSC differentiation towards salivary gland epithelial cells, the identified spots were categorized into 12 groups based on the peak time of expression during the 7 days of co-culture. The 12 categories and their corresponding spots whose expression levels were significantly changed are presented in Figure 4(p<0.05). A large proportion of the differentially expressed proteins fell within the pattern #10 showing increased expression on days 3, 5, and 7 (11 spots) and the pattern #11 showing increased expression on days 1,3,5, and 7 (12 spots) (Fig. 4). Only one protein (spot #29) exhibited a modification over time (pattern #12), which means that they have the same protein identification but the expression of one form gets stronger over time (arrowhead) while the other form shows reduced expression during the culture (arrow).

**Figure 5:**
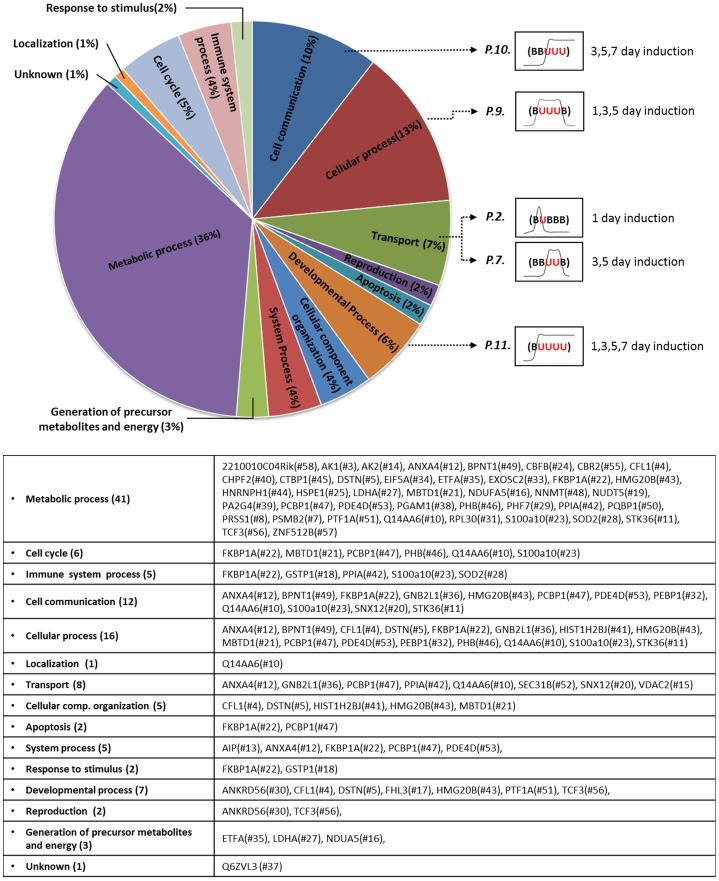
Functional categorization of proteins based on biological processes. Functional categories were generated based on the annotations of gene ontology using DAVID, PANTHER and the mouse genome informatics (MGI) GO_Slim Chart Tool. Four functional categories of cell communication, transport, regeneration and developmental process were exemplified with expression pattern profiles.

In addition, we analyzed cellular functions of identified molecules by using DAVID Bioinformatics Resources 6.7 (http://david.abcc.ncifcrf.gov) and PANTHER Protein Classification System Database (http://www.pantherdb.org) [21], [22]. Among numerous biological processes, we focused on cell communication (10%), cellular process (13%), transport (7%), and developmental process (6%) categories that are potentially important for MSC differentiation (Fig. 5). Interestingly, our analyses indicate that the pattern #10 proteins (that are induced from day 3 to day 7 of co-culture) tend to be associated with cell communication processes. The pattern #9 proteins are mainly associated with cellular processes. Additionally, the patterns #2 and #7 proteins were mainly involved in transport while the pattern #11 proteins were categorized under developmental process.

### Three transcription factors were selected from 2-DE data as novel regulators for MSC transdifferentiation

Since regulatory factors involved in differentiation play important roles in salivary gland development, we selected proteins involved in developmental processes for further analysis. Proteins involved in development include ANKRD56(#30), CFL1(#4), DSTN(#5), FHL3(#17), HMG20B(#43), PTF1α(#51), and TCF3(#56), all of which were subjected to a web-based database search of Salivary Gland Molecular Anatomy Project (http://sgmap.nidcr.nih.gov/sgmap) at the National Institute of Health/National Institute of Dental and Craniofacial Research (NIH/NIDCR). This search resulted in three proteins involved in salivary gland embryogenesis, namely high mobility group 20B (Hmg20b; spot#43), transcription factor E2a (Tcf3; spot#56) and ankyrin repeat domain-containing protein 56 (Ankrd56; spot#30). These proteins were classified as transcriptional factors according to the NIDCR database and involved in development according to our analyses in Figure 5.

**Figure 6:**
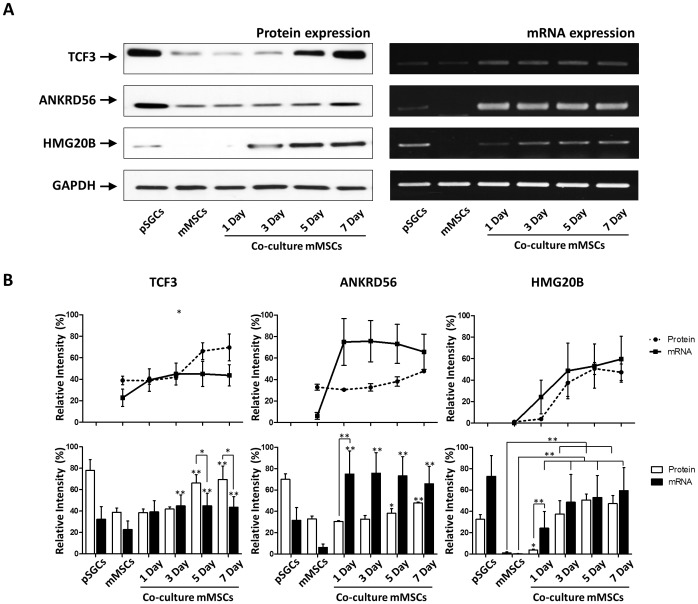
Quantitative analyses of ANKRD56, HMG20B and TCF3 expression using western blotting and RT-PCR. A) Total protein lysate and mRNA samples isolated from the pSGCs derived from the submandibular gland tissue of 4 week-old B6 mice were used as a positive control. GAPDH protein was used for a loading control. Tcf3, hmg20B and Ankrd56 proteins were analyzed in pSGCs and co-cultured mMSCs. B) Densitometer analyses of salivary acinar cell markers, such as α-AMY, M3R and AQP-5, were analyzed in three independent replicates (p<0.05, one-way ANOVA with Bonferroni post-hoc test).

To verify the expression profiles of HMG20B, TCF3, and ANKRD56, western blot analysis and gene expression profiling by semi-quantitative RT-PCR (Table S1) were performed (Fig. 6). Protein expression of TCF3 and HMG20B were highly elevated while Ankrd56 protein was moderately expressed in newly isolated pSGCs. The TCF3 and HMG20B, but not ANKRD56, showed a low level of expression in control mMSCs. After co-culturing mMSCs with pSGCs, protein expression of TCF3 was significantly increased at days 5 and 7 when compared to control mMSCs. The level of Tcf3 mRNA expression remained stable after the initial increase on day 1 (Fig. 6). In addition, protein expression of HMG20B increased gradually over time whereas gene expression was significantly increased as early as day 3 and was sustained until the end of the culture. Both gene and protein expression of Ankrd56 were significantly increased as early as day 3 and remained elevated until the end of the co-culture (Fig. 6).

## Discussion

Over the past few decades, several studies indicate numerous advantages of utilizing MSCs over other types of stem cells. They are easy to culture and expand for a prolonged period of time without transformation into cancer [23]. They also facilitate homing and engraftment of other stem cells, and tend to induce and maintain immunological tolerance [24]. A number of studies have shown that MSCs with proper stimuli can express markers associated with salivary gland epithelial cells [15], [25], [26]. However, the efficiency of differentiation was minimal and key factors that derive MSC into SGCs were not available in those studies. This lack of information prompted us to investigate for the first time these key factors by 2-DE proteomics.

To investigate the feasibility of MSC differentiation into salivary epithelial precursors *ex vivo*, we applied a co-culture system using a membrane-separated transwell. This co-culture method has been well established in various stem cell research fields. For example, Spees *et al.* found that human MSCs differentiated into epithelial cells after co-culture with damaged airway epithelial cells [27]. Zurita et al. observed differentiation of MSCs into neuronal cells upon co-culture [28]. In addition, BM-MSCs were transdifferentiated from rat [29] and human [15] salivary gland epithelial cells in an *in vitro* co-culture system. A recent study with human adipose tissue-derived MSCs indicated that these cells were capable of transdifferentiating into human SGCs *in vitro* and offered protection against radiation-induced cell damage [30]. Of note, Maria *et al*. reported that human MSCs co-cultured with pSGCs differentiated into salivary epithelial cells [15]. Presumably, soluble factors released from the salivary gland epithelial cells cross the membrane to exert their paracrine effects on the MSCs in the co-culture. This supports a notion that *in vivo* tissue regeneration may occur as a part of repair process in a particular microenvironment of tissue damage, where instructive cues for repair/regeneration become available. To our knowledge, these factors released *in vitro* as well as *in vivo* for salivary gland regeneration have not been identified.

It would be also interesting to point out that co-culture cell ratio of 1∶6 (MSC:pSGCs) and optimal amount of culture media was important in our current study to favor induction and differentiation of mMSCs into salivary epithelial cells. The possible reasons that we can speculate would be that total amount of soluble factors released from pSGCs may need to be sufficient enough to induce mMSC differentiation, which was also supported by the findings by Maria *et al*. Co-culture studies utilizing different ratios of cells used in upper and lower chambers may, in part, account for the different outcomes of MSC transdifferentiaton, which ranged from 13% to 40%. In addition, most of co-cultured mMSCs were very easy to be detached from the cell culture dish or the slide. When mMSCs were seeded on a double-coated slide with laminin and poly-D-lysine in order to enhance cell attachment, we observed that salivary epithelial marker expression in co-cultured mMSCs was markedly diminished. This suggests that optimal strength of cellular attachment to the dish/slides and sufficient amount of inductive signals in the co-culture may be critical in MSC transdifferentiation.

Our large scale proteomics approach to analyze critical proteins for mMSC differentiation involved protein separation on 2-DE with protein identification by mass spectrometry, which resulted in 58 differentially expressed protein spots. Our thorough serial examination of the spots obtained from 10 experimental replicates with statistical analyses removed the majority of false positive and negative spots. Interestingly, proteins such as AQP-5 or M3R that we observed to be differentially expressed, as detected by immunostaining, western blotting, or RT-PCR in our co-culture system, were not detected in our 2-DE analyses. This is most likely due to general insolubility of hydrophobic membrane proteins during the protein extraction process, which is an intrinsic issue commonly observed in 2-DE analysis [31]. Alternatively, proteins may have similar pI and/or molecular weights, resulting in one spot containing multiple proteins [32]. Nonetheless, we confirmed that salivary epithelial-specific markers were expressed in mMSCs during the co-culture as detected by western blotting and RT-PCR. We are currently applying a more sensitive high-throughput proteomics approach, aiming to profile a complex regulatory network of MSC differentiation and utilize the data for clinical application in conjunction with our current data.

Of those 58 proteins, Ankrd56, Hmg20b, and Tcf3 were selected based on the putative roles in the early salivary gland development. TCF3 is the most abundant TCF/LEF member in mouse ES cells [33]. It was reported that heterodimers between TCF3 and tissue-specific basic helix-loop-helix (bHLH) proteins play major roles in determining tissue-specific cell fate during embryogenesis [34]. It is also known to be closely involved in Wnt/beta-catenin signing to control self-renewal and regulates the lineage differentiation potential of ES cells toward ectoderm [35]–[38]. Interestingly, TCF3-beta-catenin interaction may indirectly affect submandibular salivary gland during mouse embryogenesis [39]. The study by Wu *et al* identified vascular integrity defect in organs such as the submandibular glands and liver in the Tcf3 knock-in mutation model, which specifically lacks Tcf3-β-catenin interaction. However, their exact functions of TCF3 in the salivary glands during development, stem cells, or MSC transdifferentiation remain largely unknown.

HMG20B is known to be expressed in various tissues [40]. Many researchers suggest that breast cancer susceptibility gene 2 and Hmg20b complex may have a role in cell cycle regulation and affect cell fate determination [41]. However, its cellular functions in cell differentiation or organ development have not been fully identified. Ankrd56 was first identified to control the yeast cell cycle regulator Swi6/Cdc10 and the drosophila signing protein Notch [42], but exact functions of Ankrd56 remain unknown. According to the NIDCR mRNA database, Ankrd56 gradually increased its expression from embryonic stage E14, whereas Tcf3 and Hmg20b were highly expressed starting at E11.5 but were slightly downregulated during the early post-natal stage of mice.

**Figure 7:**
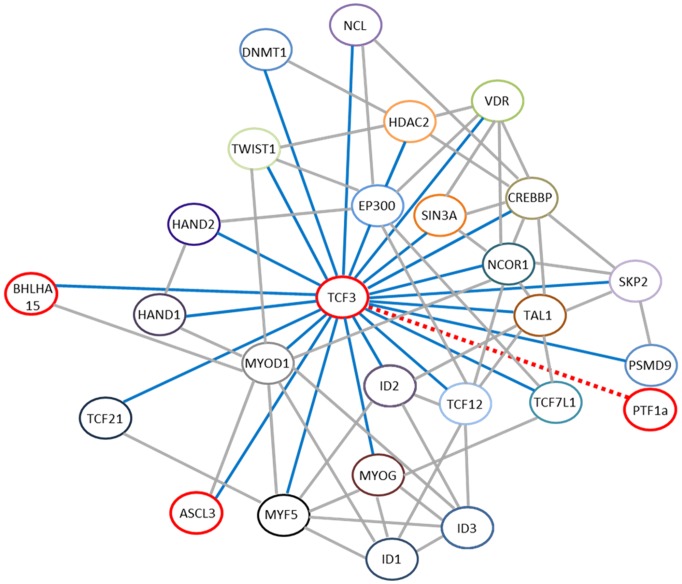
Functional network of key transcription factors during development. Based on data analysis using STRING 9.1 and WikiPathway, numerous proteins appear to be functionally associated with TCF3 during developmental processes. A dotted line indicates a potential association in function between TCF3 and PTF1α.

To further understand functional networks of ANKRD56, HMG20B, and TCF3, STRING 9.1 analysis program (http://string.embl.de) was utilized. The program neither identified nor predicted the information on the functional network and protein-protein interaction for ANKRD56 or HMG20B. However, numerous proteins were shown to be closely associated with TCF3 protein during developmental stages (Fig. 7), implying MSC differentiation is a complex process that involves numerous molecules. MIST1 (Bhlha15; bHLH family, member 15) and SGN1 (Ascl3; achaete-scute complex homolog 3) appear to be directly or indirectly involved with TCF3 (Fig. 7). In previous studies, MIST1 (Bhlha15) was speculated to affect differentiation and/or morphology of other serous exocrine cells including pancreas [43], salivary glands [44], gastric epithelium [44], [45], and mammary gland alveolar cells [46]. In addition, SGN1 (Ascl3) was associated with exocrine differentiation since it is well expressed in precursor cells in all major salivary glands [47] and known to delineate ductal cell lineage in mice [48]. In addition, PTF1α was identified as one of the 58 differentially expressed proteins by 2-DE. However, PTF1α was neither listed in the NIDCR database nor found by functional network analysis software. A dotted line in Figure 7 indicates a potential association in function between TCF3 and PTF1α based on the literature search [49], [50]. In general, studies regarding these molecules mainly utilized immunohistochemistry analyses as an experimental approach. Therefore, clear understanding of their roles in MSC differentiation for salivary gland regeneration warrants further investigation.

## Conclusions

We identified three transcription factors through 2-DE proteomics as potential regulatory molecules in driving transdifferentiation of multipotent MSCs into salivary epithelial cells. Currently, viral vectors expressing the molecules of interest are being constructed for *in vitro* MSC transduction studies as well as *in vivo* transplantation studies. With these approaches, we hope to elucidate their critical roles in salivary gland regeneration. It is worthy of note that once the glands are severely damaged as in many cases of SjS or radiation therapy patients, MSC’s ability to transdifferentiate *in vivo* would be limited due to lack of instructive cues for functional differentiation. Therefore, we hypothesize that salivary transcription factor-directed MSC differentiation may be essential in functional differentiation *in vivo*. By exploring salivary regulatory molecules by 2-DE, our current study has provided us key information towards manipulating or directing the stem cells to restore severe secretory dysfunction in patients as there are no effective therapies available currently to cure xerostomia.

## Supporting Information

Figure S1 **Hepato-STIM culture media provide more compatible condition for mMSCs and pSGCs in a co-culture system.** (A) To define the best condition for mMSC and pSGCs, cell viability in two different types of cell culture media, D-MEM/F12+Glutamax and Hepato-STIM, were evaluated by MTT assay for 1, 3, 5, 7, and 9 days without serum. (B) Isolated pSGC are seeded on a permeable transwell membrane and mMSCs are plated on a collagen-coated glass slide on the bottom of a cell culture plate.
(TIF)

Table S1 **Primer Sequences.**
(TIF)
