# Supplementary figures and images for: Identification of Regulatory Factors for Mesenchymal Stem Cell-Derived Salivary Epithelial Cells in a Co-Culture System
Source: PLoS One. 2014 Nov 17;9(11):e112158. doi: 10.1371/journal.pone.0112158 (PMC4234408; doi:10.1371/journal.pone.0112158)

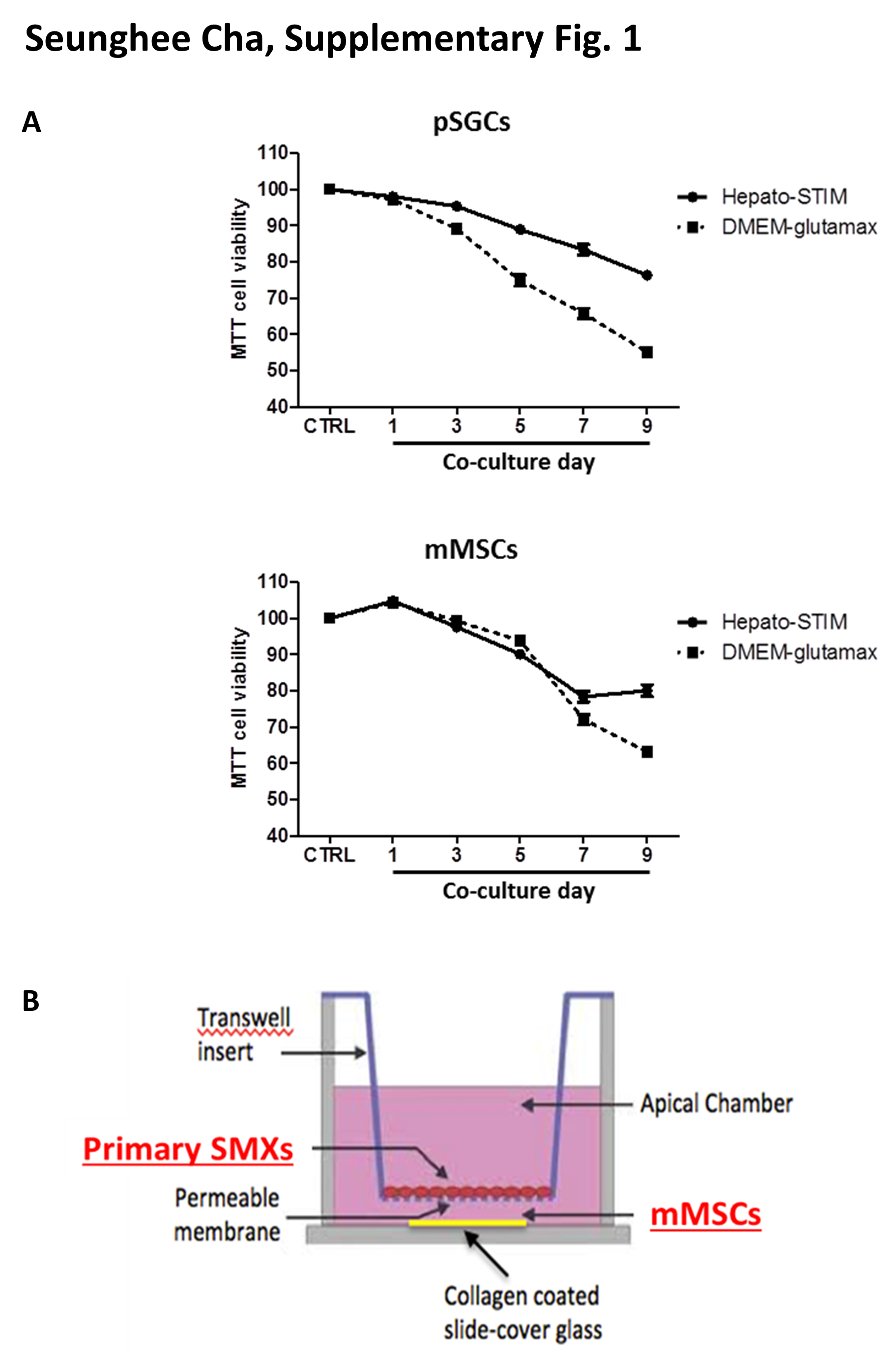

Supplement: Figure S1 — Hepato-STIM culture media provide more compatible condition for mMSCs and pSGCs in a co-culture system. (A) To define the best condition for mMSC and pSGCs, cell viability in two different types of cell culture media, D-MEM/F12+Glutamax and Hepato-STIM, were evaluated by MTT assay for 1, 3, 5, 7, and 9 days without serum. (B) Isolated pSGC are seeded on a permeable transwell membrane and mMSCs are plated on a collagen-coated glass slide on the bottom of a cell culture plate. (TIF) [file pone.0112158.s001.tif]

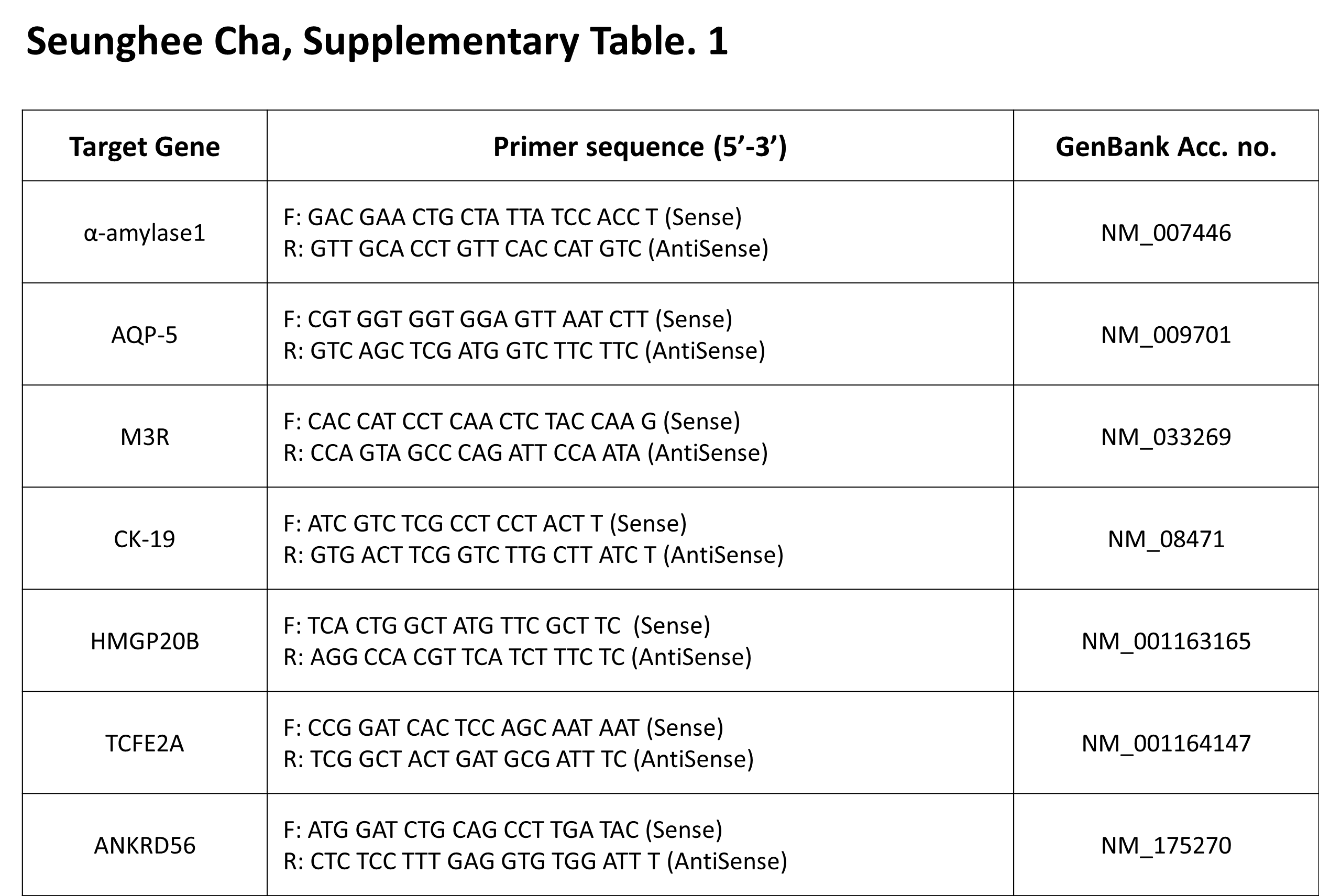

Supplement: Table S1 — Primer Sequences. (TIF) [file pone.0112158.s002.tif]
